# Prediction of prognosis and immunotherapy response in breast cancer based on neutrophil extracellular traps-related classification

**DOI:** 10.3389/fmolb.2023.1165776

**Published:** 2023-05-26

**Authors:** Jiajing Zhao, Xiaojun Xie

**Affiliations:** ^1^ General Surgery, The First Affiliated Hospital of Shantou University School of Medicine, Shantou, China; ^2^ Department of Clinical Medicine, Shantou University School, Shantou, China

**Keywords:** neutrophil extracellular traps, breast cancer, clustering, prognosis, immunotherapy response

## Abstract

Neutrophil extracellular traps (NETs), a network of DNA histone complexes and proteins released by activated neutrophils, have been demonstrated to be associated with inflammation, infection related immune response and tumorigenesis in previous reports. However, the relationship between NETs related genes and breast cancer remains controversial. In the study, we retrieved transcriptome data and clinical information of BRCA patients from The Cancer Genome Atlas (TCGA) database and Gene Expression Omnibus (GEO) datasets. The expression matrix of neutrophil extracellular traps (NETs) related genes was generated and consensus clustering was performed by Partitioning Around Medoid (PAM) to classify BRCA patients into two subgroups (NETs high group and NETs low group). Subsequently, we focus on the differentially expressed genes (DEGs) between the two NETs-related subgroups and further explored NETs enrichment related signaling pathways by Gene Ontology (GO) and Kyoto Encyclopedia of Genes and Genomes (KEGG) analysis. In addition, we constructed a risk signature model by LASSO Cox regression analysis to evaluate the association between riskscore and prognosis. Even more, we explored the landscape of the tumor immune microenvironment and the expression of immune checkpoints related genes as well as HLA genes between two NETs subtypes in breast cancer patients. Moreover, we found and validated the correlation of different immune cells with risk score, as well as the response to immunotherapy in different subgroups of patients was detected by Tumor Immune Dysfunction and Exclusion (TIDE) database. Ultimately, a nomogram prognostic prediction model was established to speculate on the prognosis of breast cancer patients. The results suggest that high riskscore is associated with poor immunotherapy response and adverse clinical outcomes in breast cancer patients. In conclusion, we established a NETs-related stratification system that is beneficial for guiding the clinical treatment and predicting prognosis of BRCA.

## 1 Introduction

Breast cancer is one of the highest incidence of malignant tumors around the world (Lei, et al., 2021; Qiu, et al., 2021; Sung, et al., 2021). It occupies approximately 30% of new cancer cases, making it a serious threat to women’s health (Hong, et al., 2018; McDonald, et al., 2016). Drug resistance, recurrence and metastasis are verified as the three main factors affecting the prognosis of breast cancer patients (Harbeck, et al., 2019). The conventional view considers that breast cancer is not an immunogenic tumor. However, immunotherapy has become a vital treatment since breast cancer was confirmed as an immunogenic tumor recurrently (Vranic, et al., 2021). With the development of immune agents, immunotherapy combined with chemotherapy for triple-negative breast cancer (TNBC) with PD-L1 overexpression has been approved as a first-line treatment (Franzoi, et al., 2021). At the same time, the marketing of some monoclonal antibodies has proved that immunotherapy can significantly improve the survival of HER2^+^ breast cancer patients. In addition, immune checkpoint inhibitors (ICI) such as Atezolizumab and Pembrolizumab have shown promising results in the treatment of TNBC (Henriques, et al., 2021). Therefore, personalized selection of immunotherapy based on different breast cancer subtypes is extremely important to improve the prognosis of breast cancer patients.

It is well known that human neutrophils are the most abundant leukocyte type and an essential component of the host response to different pathogens (Poto, et al., 2022). Recurrently, significant advances have been made in the understanding of the role of neutrophils in immune system regulation, pathogen clearance, and disease pathology (Papayannopoulos, 2018). Studies in humans and mice have shown that there are two types of neutrophils: anti-tumor N1 neutrophils and native N2 neutrophils (Fridlender, et al., 2009; Fridlender and Albelda, 2012) Most clinical evidence supports the idea that neutrophils promote rather than inhibit progression (Templeton, et al., 2014). Neutrophil elastase (NE) belongs to the serine protease family and is mostly expressed in polymeric neutrophils (PMN) (Pulford et al., 1988; Fouret, et al., 1989; Molldrem et al., 2002). During neutrophil degranulation or neutrophil extracellular trap (NETs) formation, it is released into the extracellular space, which called NETosis. NETs are composed of DNA-histone complexes and proteins secreted by activated neutrophils (Masucci, et al., 2020). Studies has shown that it is involved in the progression and metastasis of cancer, both in animal and in cancer patients. What’s more, the release of NETs occurring during neutrophil regulatory death named NETosis, is a pivotal functional pathway by which neutrophils mediate toxic injury (Zhu, et al., 2022). NETosis is believed to be a source of autoantigens and to maintain the maintenance of the inflammatory environment that promotes autoimmune diseases (Klopf, et al., 2021). Interestingly, NETs play a different role in pan cancer. Higher NET scores were connected with favorable survival of some kinds of cancer, such as kidney renal clear cell carcinoma (KIRC) and lung adenocarcinoma (LUAD) (Shen, et al., 2022). However, NETs have also been reported to be associated with better survival in patients with head and neck squamous cell carcinoma (Millrud, et al., 2017). At present, predicting the prognosis of different tumors by scoring NETs-related genes datasets is still controversial. The effect of NETs-related genes on prognosis in BRCA patients remains unclear. Moreover, there is few reports about the effects of NETs-related genes on tumor-related immune cell infiltration and prognosis of breast cancer.

In this study, we aimed to search for NETs-related biomarkers and construct a NETs risk model to predict the tumor-associated immune microenvironment, prognosis and response to immunotherapy in BRCA patients. The research could help doctors to make treatment decisions for different types of BRCA in the future.

## 2 Materials and methods

### 2.1 Database and data preprocessing

Firstly, we retrieved the RNA-seq transcriptome data (FPKM format) of a total of 1,211 samples from TCGA database, and then converted the FPKM (Frequencies Per Kilobase per Million) format RNA-seq data into TPM (scripts per million reads) format and following converted the log2 to obtain the matrix of samples and gene expression. It is worth mentioning that the samples with missing expression value, short overall survival time (overall survival equals zero and missing clinical information were excluded from this study. Finally, 1,093 samples were included in this research, including 107 normal paracancerous tissue and 986 tumor tissues. The complete TCGA cohort clinical information is summarized in Supplementary Table S1. Most of the patients were female (99.1%), and the median age was 58 (48, 67). The TCGA cohort data are reliable because the TCGA-BRCA data adhere to strict follow-up criteria. Additionally, we downloaded the microarray data (accession number is GSE21653) containing 266 breast cancer samples from the Gene Expression Omnibus (GEO) database as the validation set of this study after the same pre-processing as the TCGA database training set. Additionally, the clinical data in the two data sets are indispensable in this study.

### 2.2 Validation of hub genes

To further confirm the difference of hub gene expression between breast cancer tumor tissues and normal breast tissues, we downloaded the breast tissue sections of BRCA and healthy people from the Human Protein Atlas (HPA) database. The immunohistochemical result of LTF (Antibody:CAB008646), ENO1(Antibody: CAB080034), LCP1(Antibody: HPA019493) and AZU1 (Antibody: HPA075964) were shown. Additionally, in order to analyze the variations of various hub genes across tumor tissues and normal tissues, we acquired the total protein expression data of hub genes from the UALCAN database.

### 2.3 NETs-related gene set and consensus clustering

In our research, we obtain 23 genes of NET protein released by human neutrophils identified in previous studies (Gardinassi, et al., 2017; Wither, et al., 2018; O'Donoghue, et al., 2013). Since DEFA3 is not retrieved in the RNA-Seq matrix, the expression quantity of 22 NETs-related genes in tumor tissues was extracted for further analysis. ConsensusClusterPlus tool in R software was applied for consensus cluster analysis (Wilkerson and Hayes, 2010). Partitioning Around Medoid (PAM) clustering with 1-pearson correlation distance was performed and 80% of the samples were repeated for 1,000 times to ensure the stability of the results. An empirical cumulative distribution function plot was used to determine the optimal number of clusters. According to the evaluation of the area under the line of the cumulative distribution function (CDF) curve, when the increase of K value is the gradual increase of the area under the line of the CDF curve, we need to keep the area under the line as large as possible under the premise, according to the evaluation of the CDF delta downward trend, try to keep the delta downward the slowest, combine the above two factors compromise to choose the number of clusters. We determined the ideal clusters in molecular subtypes related to NETs (k = 2). The “pheatmap” tool in R is used to generate the cluster graph.

### 2.4 Differential gene expression and function enrichment analysis

The “limma” package in R (Ritchie, et al., 2015) was applied to analyze the differential gene expression between tumor samples and paracancerous tissue samples to obtain the differential gene expression between different tumors and normal tissues. In order to exclude false-positive TCGA data, we set the *p* values < 0.05, | log2FC | > 1.5 genes for DEGs to control the number of differential genes and reduce the likelihood of false-positive results. Subsequently, GO and KEGG analyses were performed to compare the signaling pathways and biological processes between the upregulated NETs group and the downregulated NETs group. For gene set functional enrichment analysis, we used KEGG test API. Up-to-date KEGG pathway gene annotations were obtained and genes were mapped to background sets. The R package clusterprofiler (v.3.14.3) was used for enrichment analysis, and the gene set enrichment results were obtained. The minimum gene set was set at 5, and the maximum gene set was set at 5,000. *p* < 0.05 and FDR < 0.25 were considered statistically significant.

### 2.5 Gene Set Enrichment Analysis (GSEA)

For GSEA (Subramanian, et al., 2005), we downloaded the GSEA software (v.3.0) from the website and defined the gene rank in advance and from Molecular Signatures database (MsigDB) (Liberzon, et al., 2011), “C2. Cp. Reactome. V7.4. Symbols” [29]. The “Gmt” subset was powered to evaluate related pathways and molecular functions. According to the predetermined gene rank, the smallest gene set was 5 and the largest gene set was 5,000. Finally, *p* < 0.05, FDR < 0.25 were statistically significant.

### 2.6 Prognosis survival analysis

R package “maxstat” (v.0.7–25) was utilized to determine the optimal cutoff value of riskscore. We determined the optimal cut-off value of RiskScore by setting the minimum sample size to be greater than 25% and the maximum sample size to be less than 75%, and then determining the optimal cut-off value. Patients were divided into high and low categories based on this result. Furthermore, we analyzed the prognosis difference between the two subgroups using the R package “survival” to evaluated the significance of the prognosis between different groups of samples by the Log-rank test. We concentrated on the prognosis difference. In addition, Univariate and Multivariate Cox regression analysis were used to independent prognostic factors affecting overall survival (OS) of BRCA patients.

### 2.7 NETs-related risk signature model and immunotherapy prediction

Least absolute shrinkage and selection operator (LASSO) regression analysis is a linear regression method using L1-regularization. Compared with the traditional Cox regression analysis, LASSO Cox regression analysis can solve collinearity problem. The R package “glmnet” was used for LASSO Cox regression analysis of status, survival time, survival and RNA-seq data. Besides, we set a 10-fold cross-check to obtain the optimal model. Riskscore was calculated according to the formula: Riskscore = $\sum_{i=1}^{n}\left(Coefi\;*\;Expi\right)$ . In addition, Tumor Immune Dysfunction and Exclusion (TIDE) was applied to detect BRCA patient’s response to immunotherapy (http://tide.dfci.harvard.edu/). TIDE database is an analytical tool that effectively predicts the response to immune checkpoint inhibition based on two major tumor immune escape mechanisms: T-cell exhaustion and T-cell infiltration.

### 2.8 Tumor associated immune cell landscape of NETs-Related genes

Based on our expression profile, we used the R software package “IOBR” to select “ESTIMATE” method to calculate the estimate score, immune score and stromal score (Yoshihara et al., 2013). Besides, CIBERSORT was powered to compute the scores of 22 kinds of immune infiltrating cells between the two NETs subgroups (Newman, et al., 2015). The results of tumor immune cell infiltration are presented by landscape map.

R package “ggplot2” was utilized for visualization of stack, violin and bar plots in immune cell landscape. Furthermore, Sangerbox tool was conducted to assist in describing the immune cell landscape (Weitao, et al., 2022).

### 2.9 Somatic mutation

For somatic mutation, we downloaded “maf” format data for somatic mutations in breast cancer patients from the TCGA database. “Maftools” package in R was applied to generate waterfall map for the visualization of somatic mutant genes.

### 2.10 Statistics

All statistical analysis was conducted using R software (v.4.2.1). Sangerbox, an online analysis and auxiliary drawing tool, is used to visualize some of the images. The TCGA-BRCA training set and the GSE21653 test set were standardized by zero-mean normalization. Kaplan-Meier analysis was powered to analyze the difference in survival outcomes between NETs-high group and NETs-low group. Log-rank test was used to calculate statistical significance between the two groups. In addition, the receiver operating characteristic (ROC) curve was applied to measure prognostic prediction performance by area under the curve (AUC) of NETs-associated genes and the prediction model. *p*-value < 0.05 was considered statistically significant.

## 3 Result

### 3.1 Consensus clustering of NETs-Related subgroups

**FIGURE 1:**
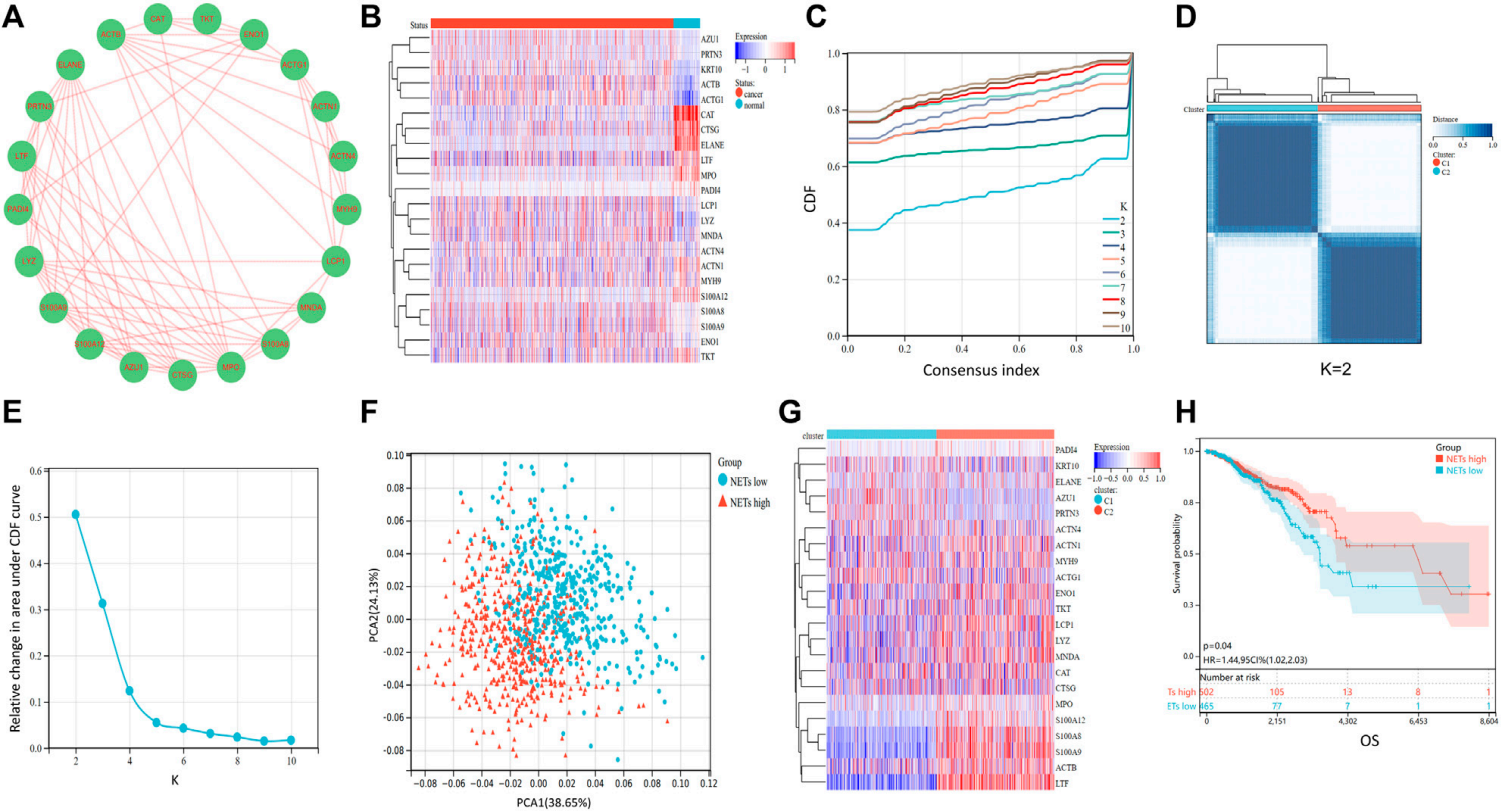
Consensus clustering of NETs-related subgroups **(A)** Protein-Protein Interaction Network (PPI) of NETs-related genes was generated by Cytoscape software **(B)** Heatmap of NETs-related gene expression between normal and tumor tissues **(C)**The relative change of the area under the cumulative distribution function (CDF) curve for k = 2 to 10 **(D)** A heatmap shows the consensus clustering solution of 22 NETs-related genes in breast cancer samples when k = 2 **(E)** Delta area reflects relative change in area under the CDF curve when k = 2 to 10 **(F)** PCA analysis of two NETs-related subgroups in TCGA database **(G)** Heatmap of NETs-related gene expression between C1 and C2 subgroups **(H)** Kaplan-Meier curve of OS prognosis between NETs high and NETs low subgroups.

NETs related genes in the previous literature reports summarize (Shen, et al., 2022). Based on the STRING database, Cytoscape (https://cytoscape.org/) was utilized to generate the Protein-Protein Interaction Network (PPI) of NETs-related genes to further understand the associations between NETs-related genes (Figure 1A). To understand the differences in gene expression between tumor and normal tissues, we generated a heat-map to analyze differentially expressed genes between tumor and normal tissues. Most of the NETs-related genes are downregulated in BRCA compared to normal tissues, including CAT, CTSG, ELANE, LTF, MPO, PADI4, ACTN1, MYH9, S100A12, TKT. A few of NETs-related genes are highly expressed in breast cancer, such as ACTB, ACTG1, KRT10 (Figure 1B). Aim to investigate differences among different NETs expression subgroups, consensus clustering was applied to divide BRCA patients into two clusters (k = 2). After k-medoids clustering, the TCGA-BRCA cohort was divided into two NETs-related subgroups with different expression patterns (C1 and C2) (Figure 1D). CDF delta area plot was generated to evaluate the area under the distribution curve and sample consistency of different number of subgroups (k = 2–10) (Figure 1C, E). Besides, principal component analysis (PCA) of TCGA training set showed that different NETs related subsets were independent of each other (Figure 1F). In order to analyze the expression of NETs related genes between C1 and C2 subgroups, 22 NETs-related genes were visualized by heatmap (Figure 1G). The results showed that the expression of NETs related genes was higher in cluster C2 than in cluster C1. Therefore, we define C1 cluster as NETs low group and C2 cluster as NETs high group. Furthermore, Kaplan-Meier plot was powered to evaluate the overall survival time between the two subgroups. Interestingly, the NETs high group was associated with favorable clinical prognosis and the NETs low group was associated with adverse clinical prognosis in the TCGA cohort (Figure 1H).

### 3.2 Differentially expressed genes and gene set enrichment analysis between NETs subgroups

**FIGURE 2:**
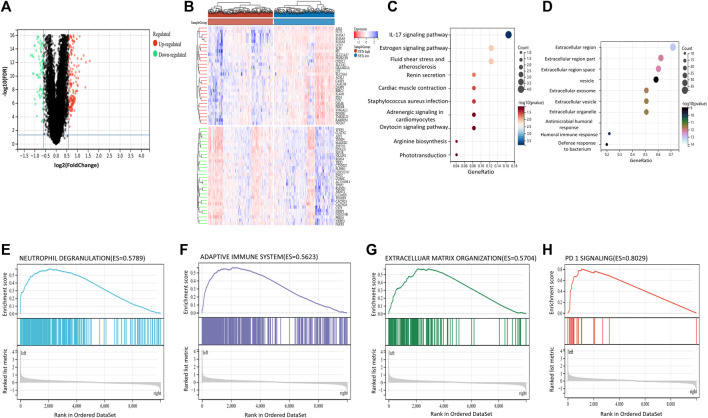
Differentially expressed genes and Gene Set Enrichment Analysis between NETs subgroups (**A, B**) Volcano plot and heatmap are used to show differentially expressed genes (DEGs) between NETs high and NETs low subgroups (**C, D**) KEGG and GO analysis of DEGs between two subgroups (**E, H**) GSEA analysis respectively suggested that upregulated DEGs in neutrophil degranulation (**E**), adaptive immune system (**F**), extracellular matrix organization (**G**) and enrichment of PD-1signaling pathway (**H**).

According to the above results, high expression of NETs-related genes suggests favorable prognosis, while low expression of NETs suggests poor prognosis. Therefore, we aimed to further explore the differences in gene expression between NETs subgroups and the related signaling pathways enriched by DEGs. The “limma” package in R software was used to analyze the DEGs between NETs high group and NETs low group. Volcano plot was generated to show DEGs, including 293 significantly upregulated genes (|log FC|>1.5) and 108 downregulated genes (|log2FC|<1.5) (Figure 2A). Representative upregulated and downregulated genes are shown in a heatmap (Figure 2B). KEGG analysis showed that NETs-related DEGs was mainly associated with IL-17 signaling pathway, various hormones (including renin, epinephrine, oxytocin, estrogen) signaling pathway, myocardial contraction and bacterial infection (Figure 2C). GO analysis showed that DEGs are related to extracellular vesicle, extracellular exosome, extracellular organelles, bacterial defense and human immune system (Figure 2D). These results suggest that these NETs-related DEGs are involved in the regulation of extracellular substance composition and human immune system. In order to further explore the signaling pathways associated with NETs and human health, GSEA was applied to analyze the signaling pathways of differential gene enrichment between NETs high group and NETs low group. GSEA analysis results showed that there were differences in the enrichment of gene sets among NETs subgroups. High expression of NETs-related genes was associated with neutrophil degranulation, adaptive immune system, extracellular matrix organization and PD-1signaling pathway (Figure 2E–H).

### 3.3 Tumor microenvironment landscape in BRCA patients

**FIGURE 3:**
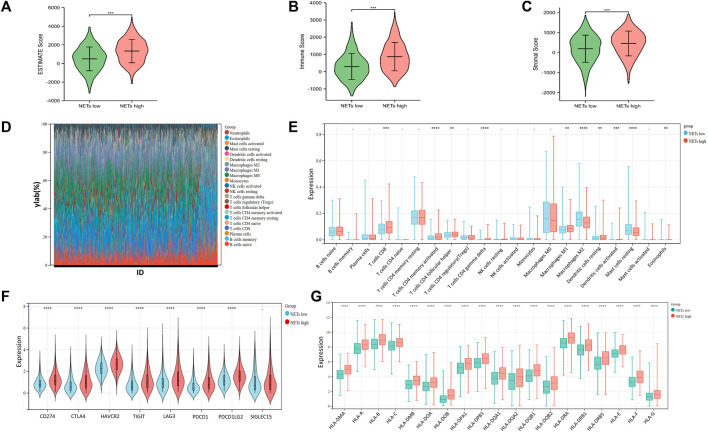
Tumor microenvironment landscape in BRCA patients **(A–C)** Violin plot is generated to show the estimate score **(A)**, immune score **(B)** and somatic score **(C)** between NETs high and NETs low subtypes **(D, E)** Stack and box plots were used to represent 22 types of tumor-related immune cells between the two NETs subgroups in BRCA **(F–G)** Violin plot and box plot showed the expression of immune checkpoint related genes **(F)** and HLA genes **(G)** between two different NETs subgroups, respectively (*p < 0.05, **p < 0.01, ***p < 0.001. ****p < 0.0001).

The infiltration of immune cells in the tumor immune microenvironment of BRCA has been confirmed to play an important role in tumor genesis, progression, invasion and drug resistance, which is related to the clinical prognosis of BRCA patients (Yuan, et al., 2021). To understand the tumor microenvironment landscape between different subtypes of NETs in breast cancer patients, ESTIMATE algorithm was performed to determine the estimated score, immune score, and stromal score between the two groups, respectively (Figures 3A–C). The results showed that the estimated score, immune score, and stromal score of the NETs high group were higher than those of the NETs low group, suggesting that the NETs high group has a favorable tumor microenvironment, which may be related to a beneficial clinical prognosis. Additionally, we generated stacking plot and box plots by the CIBERSORT algorithm for visualization of 22 kinds of tumor associated immune cells (Figure 3D, E). From the results, most of the NETs high subgroup had a higher level of immune cell infiltration than NETs low group. Specifically, CD8T cell, CD4T cell memory activated cell follicular helper, γδT cell, macrophage M1, dendritic cell, and mast cell. Immune checkpoint inhibitors are important targets of immunotherapy. Violin plots were generated to determine the expression of immune checkpoint genes between NETs subtypes (Figure 3F). Obviously, the expression of most immune checkpoint related genes in NETs high expression group was higher than that in NETs low expression group, such as CTLA4, CD274, HAVCR2, TIGIT, PDCD1, PDCD1LG2, LAG3. Furthermore, box plots were used to demonstrate the expression quantity of human leukocyte antigen (HLA) genes between NETs subgroups (Figure 3G). The results showed that HLA related genes in the NETs high group were significantly upregulated compared with those in the NETs group.

### 3.4 Somatic mutation and construction of NETs-related risk signature model

**FIGURE 4:**
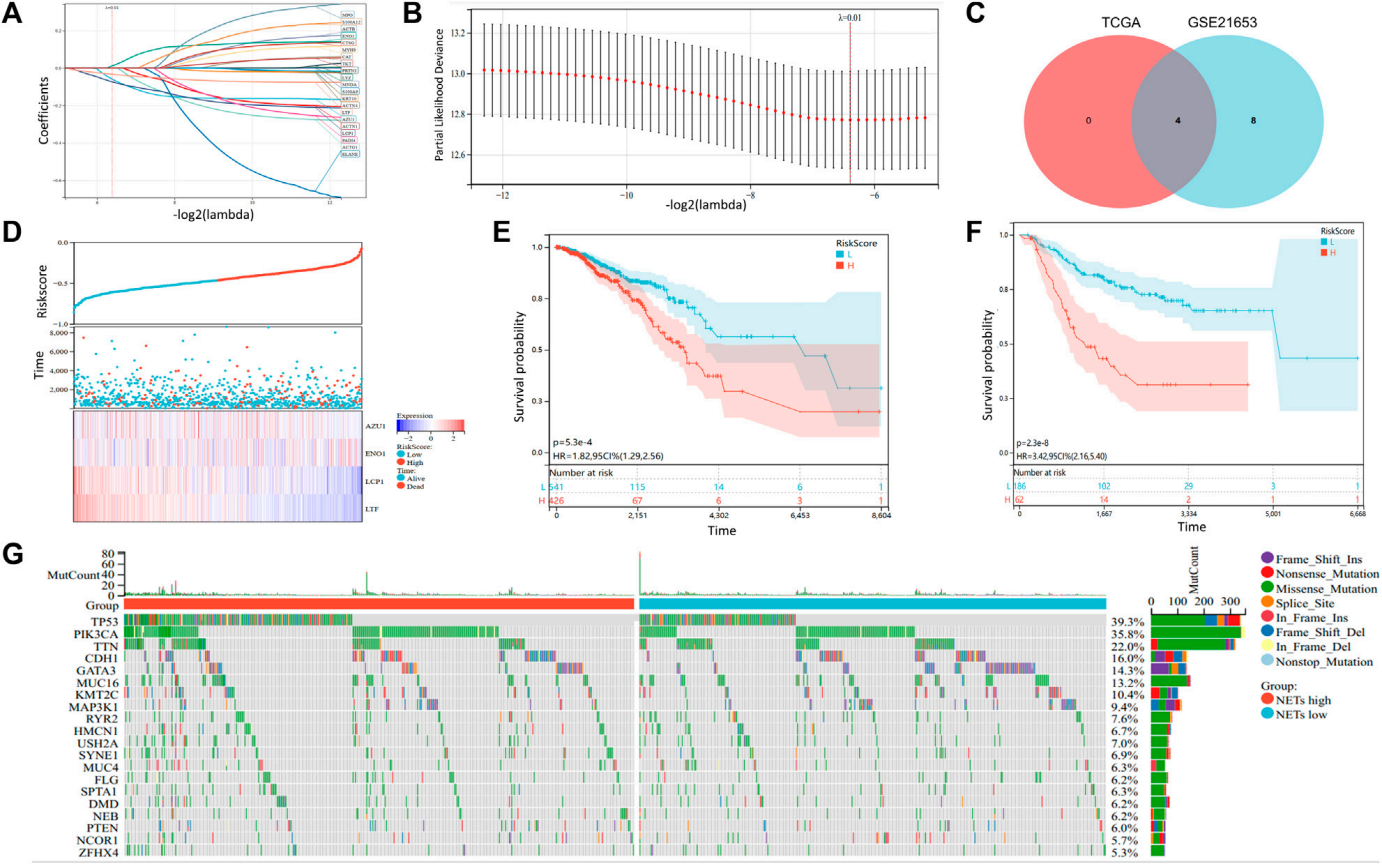
Somatic mutation and construction of NETs-related risk signature model **(A, B)** LASSO Cox regression analysis revealed four NETs-related genes associated with the OS prognosis **(C)** Venn diagram was generated to show 4 NETs-related genes related to the prognosis of OS **(D)** Survival time and status distribution heatmap of 4 NETs genes associated with OS prognosis **(E–F)** Kaplan-Meier curves associated with OS prognosis of the TCGA cohort **(E)** and the GSE21653 **(F)** cohort in different risk score subgroups **(G)** Waterfall plot of somatic mutations in the TCGA cohort.

In order to further screen NETs genes related to prognosis, we performed LASSO analysis on 22 NETs-related genes. To obtain the optimal model. The Lambda value was set to 0.012 and we finally identified four genes: LTF, LCP1, AZU1 and ENO1 (Figures 4A, B). To ensure reliable hub genes, we screened common hub genes from LASSO Cox regression analysis of GSE21653 validation set and TCGA training set and drew Venn diagram (Figure 4C). Finally, we obtain the calculation formula of Riskscore: Riskscore= ((0.012*ENO1 exp) + (−0.059*AZU1 exp) + (−0.073*LCP1 exp) + (−0.033) *LTF exp). Moreover, a heatmap was generated to evaluate the relationship between Risk Score and overall survival (Figure 4D). The results showed that high Risk Score predicted a poor prognosis. Low Riskscore indicates a favorable prognosis. In addition, we also use the GSE21653 verification set to verify this result (Figure 4E). Furthermore, heatmaps were generated to show the distribution between risk score and OS event between NETs subtypes (Figure 4F). The accumulation of somatic mutations is a feature of malignant tumors (Shibata, et al., 2021. Aim to further understand the somatic mutation of BRCA, we download the mutation omics data of BRCA from TCGA database to generate a waterfall map (Figure 4G). The results showed that TP53, PIK3CA and TTN were the main mutation genes, accounting for 39.3%, 35.8% and 22.0% in total. The incidence of TP53 and PIK3CA mutations in NETs high expression group was higher than that in NETs low expression group.

### 3.5 Association of risk signature with tumor associated immune cells and immunotherapy prediction of NETs subgroups

**FIGURE 5:**
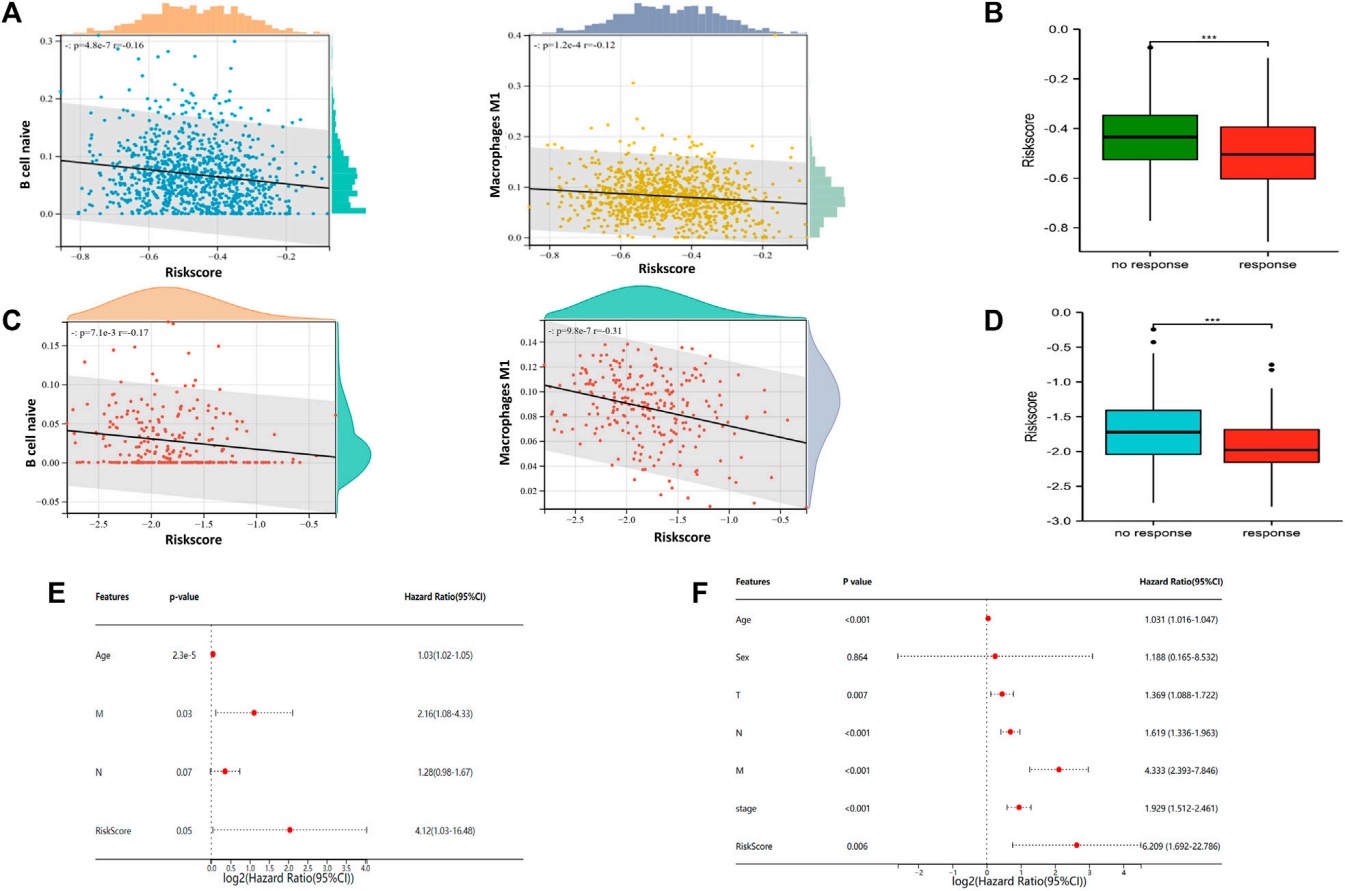
Association of riskscore signature with tumor associated immune cells and immunotherapy prediction of NETs subgroups **(A)** Scatter plot of risk score correlation between native B cell and macrophage M1 in TCGA cohort **(B)** Box plots are used to represent risk score related immunotherapy responses predicted by the TIDE database in TCGA cohort **(C)** Scatter plot of risk score correlation between native B cell and macrophage M1 in GSE21653 cohort **(D)** Box plots are used to represent risk score related immunotherapy responses predicted by the TIDE database in GSE21653 cohort **(E–F)** Forest plots were generated to show clinical features and risk score associated with OS in univariate regression analysis **(E)** and multivariate regression analysis **(F)** (***p < 0.001).

To explore the relationship of riskscore with tumor immune cell infiltration, scatter plot was generated to the correlation of riskscore with immune cells for visualization in TCGA training set (Figure 5A). Similarly, we obtained same results in the GSE21653 dataset (Figure 5C). The results showed that riskscore was negatively correlated with B cell naive and macrophage M1. The higher the riskscore, the less B cell naive and macrophage M1. Additionally, we predicted the response of BRCA patients to immunotherapy by in TIDE Database (Figures 5B,D). All samples were divided into immunotherapy-response group and non-response group. Interestingly, we found a significantly lower riskscore in the group that responded to immunotherapy than in the group that did not respond to immunotherapy. This suggests that patients with lower riskscore would benefit more from immunotherapy. Univariate and Multivariate Cox regression analysis forest plots were drawn to analyze the risk factors associated with prognosis (Figures 5E,F). The results indicated that age and riskscore were significantly associated with prognosis in both Univariate and Multivariate Cox regression analyses.

### 3.6 Construction of prognosis related risk model

**FIGURE 6:**
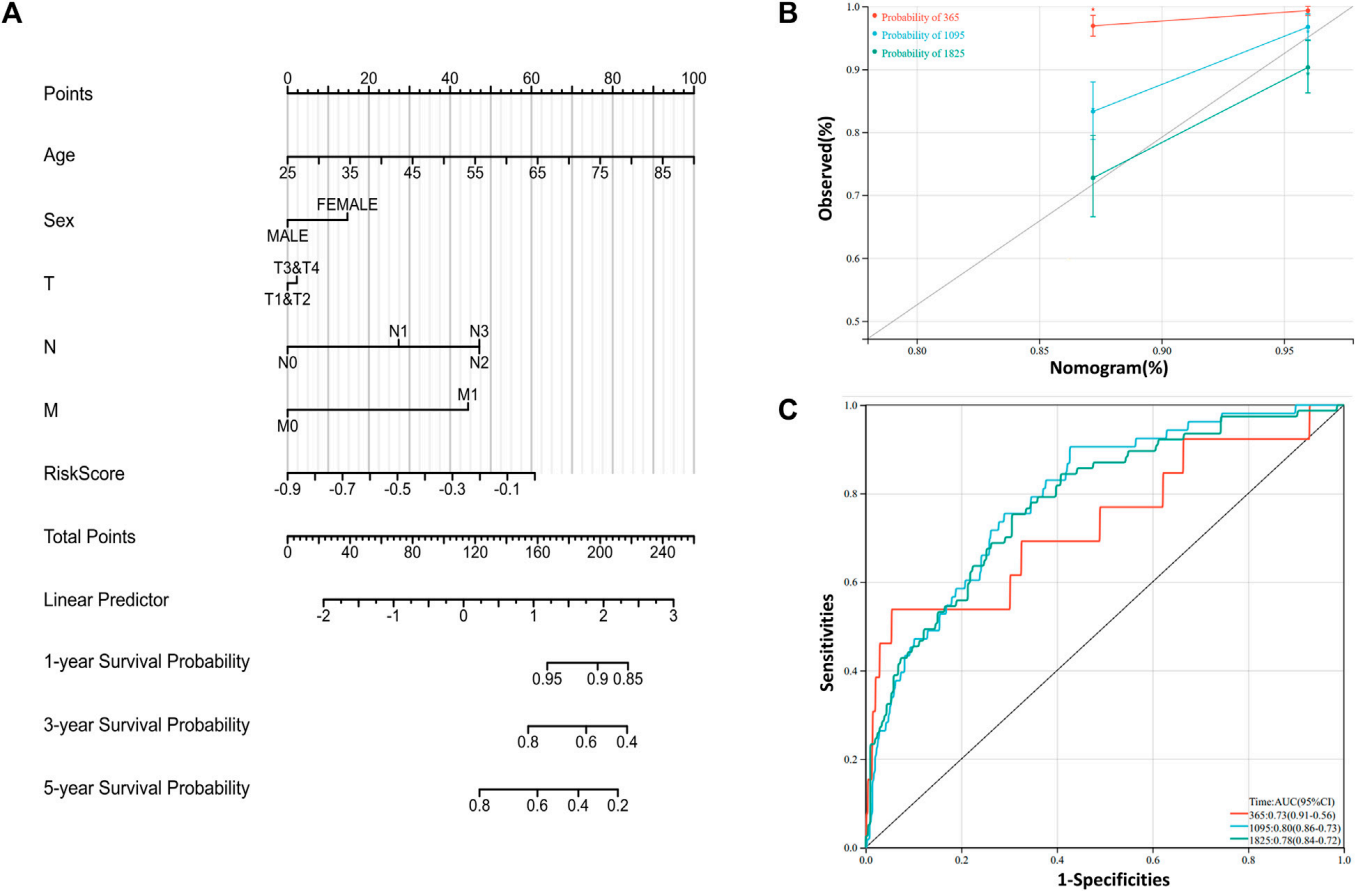
Construction of prognosis related risk model **(A)** Nomogram is generated to predict 1-,3-, and 5-year overall survival in patients with BRCA patients **(B)** Calibration curves were powered to evaluate the reliability of the model **(C)** ROC curves were drawn to evaluate the accuracy of the model in predicting 1-, 3- and 5-year overall survival.

To further understand and predict the overall survival of BRCA patients, a nomogram was utilized to calculate the prognosis of BRCA patients (Figure 6A). Total score was calculated according to the scores corresponding feature of each patient, including age, gender, lymph node metastasis, tumor size, distant metastasis and riskscore to speculate on 1-, 3-, and 5-year overall survival rates of BRCA patients. For example, a 45-year-old woman has a tumor with TMN grade (T2N2M1) and a calculated risk score of −0.2. Then the patient calculated a total score of 184. According to the risk prediction model, the 1 −, 3 −, and 5-year survival rates were 92%, 60%, and 38%, respectively. The prediction model was adjusted to observe the accuracy of the model in predicting the prognosis of breast cancer (Figure 6B). Additionally, ROC curves were generated to evaluate the reliability of this prediction model (Figure 6C). From the results, the area under ROC curve of 1−, 3- and 5-year are 0.73, 0.80 and 0.78, respectively. The consistency index (C-index) of the prediction model is 0.75, and the reliability of the model is medium.

### 3.7 Validation of hub genes between tumor tissue and normal tissue

**FIGURE 7:**
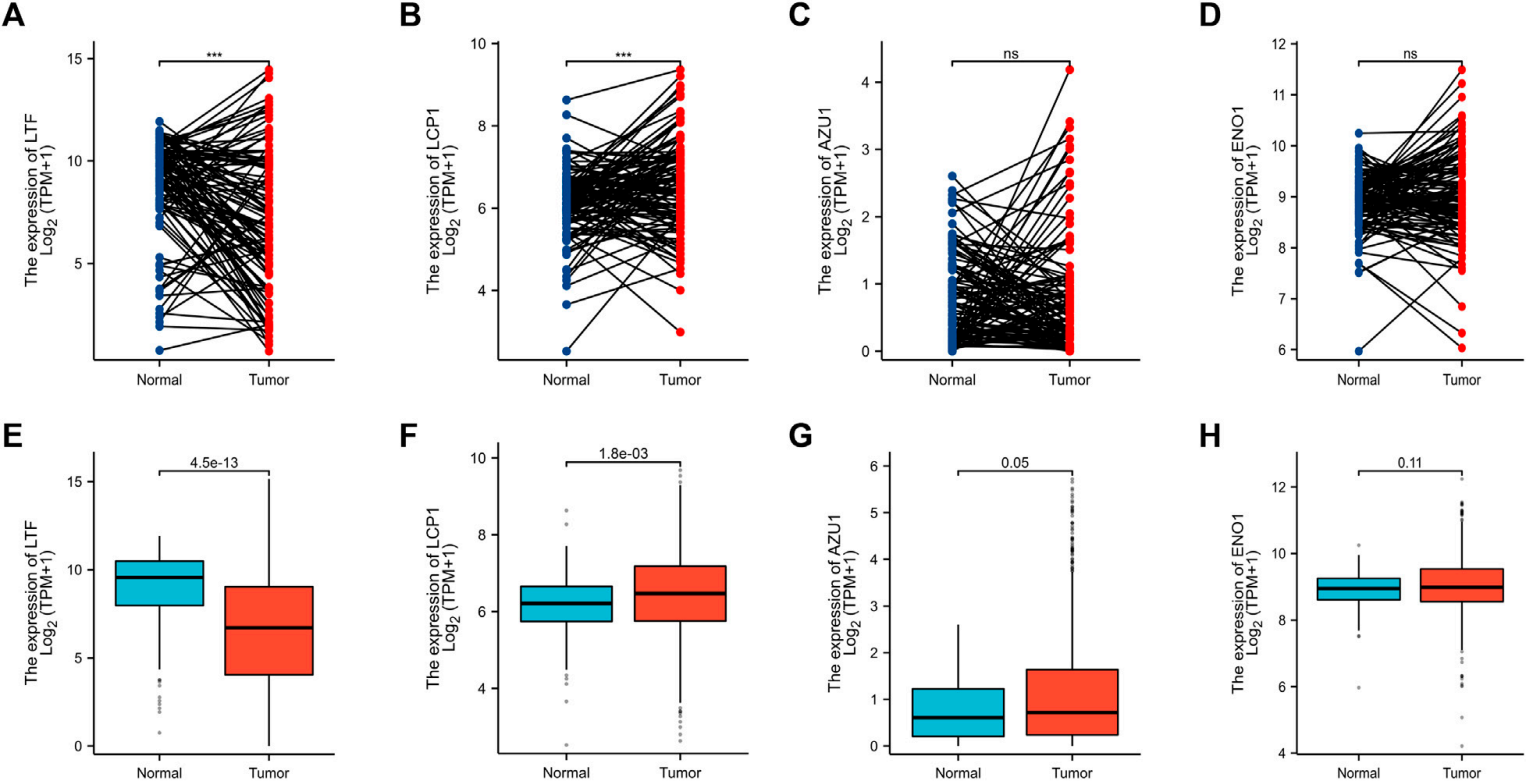
Validation of hub genes **(A–D)** Expression levels of LFT **(A)**, LCP1 **(B)**, AZU1 **(C)**, and ENO1 **(D)** in paired breast cancer samples from the TCGA database. Expression levels of LFT **(E)**, LCP1 **(F)**, AZU1 **(G)**, and ENO1 **(H)** in unpaired breast cancer samples from the TCGA database (***p < 0.001).

**FIGURE 8:**
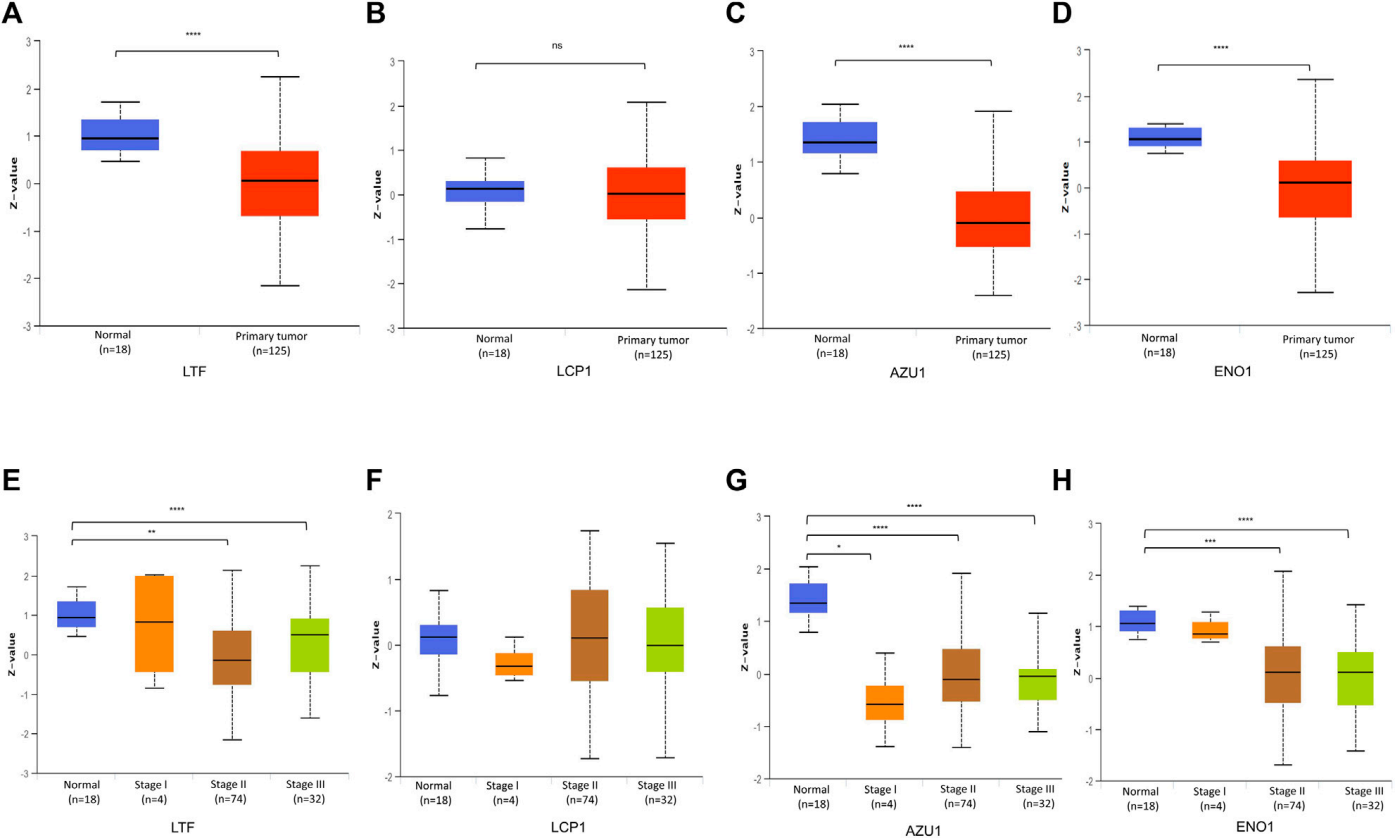
Validation of hub gene protein level **(A–D)** Total protein levels of LFT **(A)**, LCP1 **(B)**, AZU1 **(C)**, and ENO1 **(D)** in paired breast cancer samples from the CPTAC database **(E–H)** Expression of LTF **(E)**, LCP1 **(F)**, AZU1 **(G)**, and ENO1 **(H)** in various grades of BRCA patients (*p < 0.05, **p < 0.01, ***p < 0.001. ****p < 0.0001).

**FIGURE 9:**
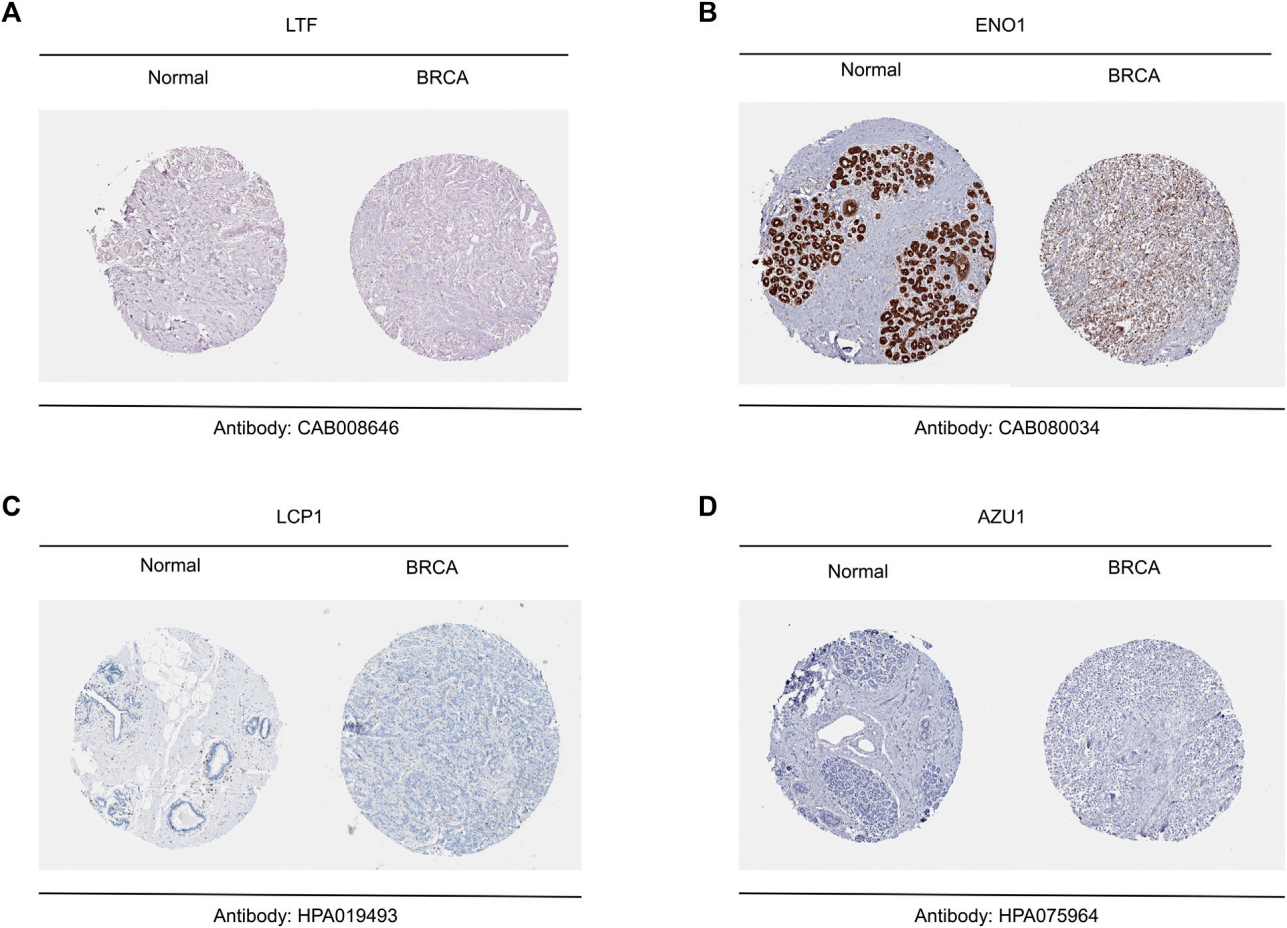
Validation of hub genes between breast tumor and normal tissue **(A–D)** Representative results of immunohistochemistry for LTF **(A)**, ENO1 **(B)**, LCP1 **(C)** and AZU1 **(D)**.

**FIGURE 10:**
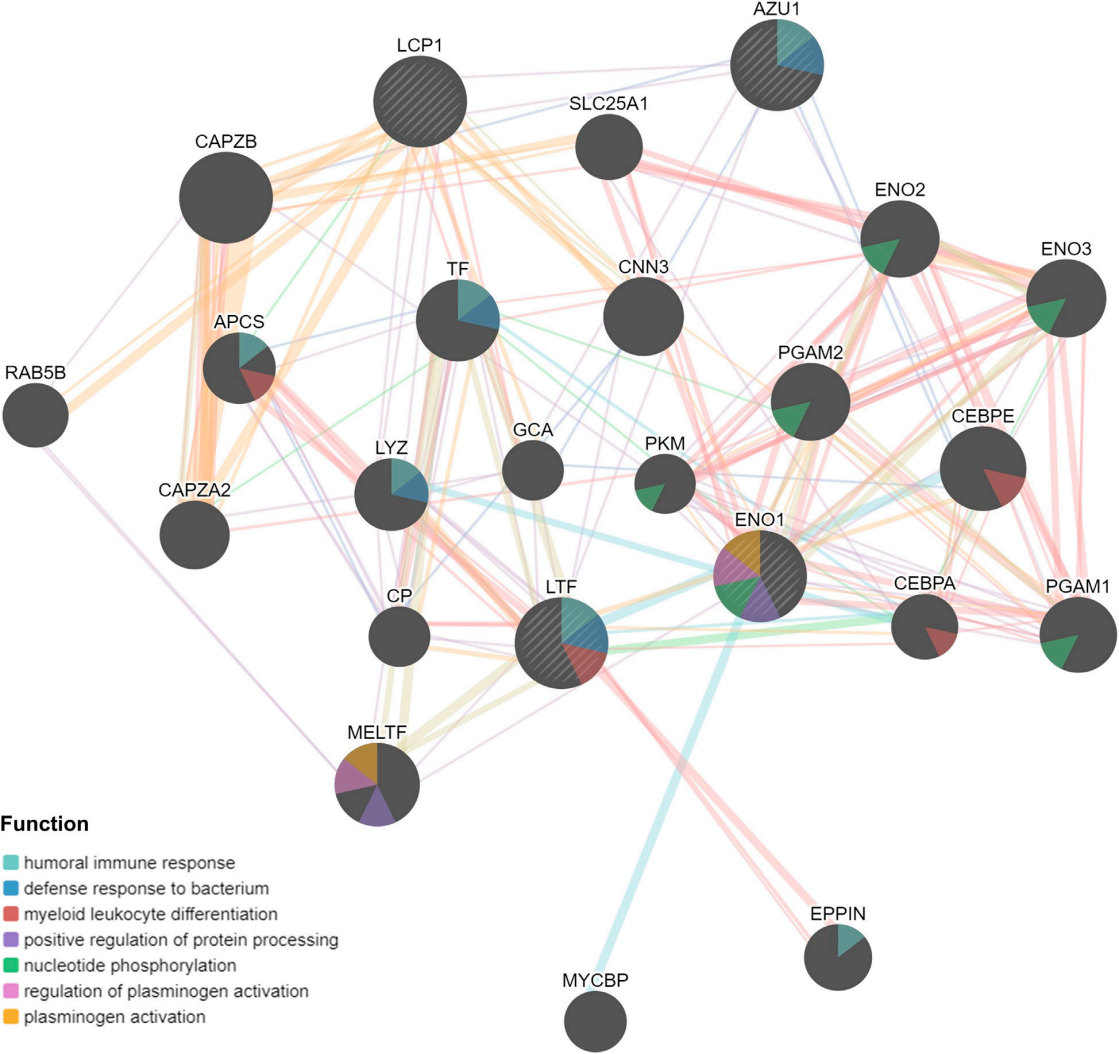
PPI networks of *LTF*, *LCP1*, *AZU1*, and *ENO1* from GeneMANIA database.

Based on above results, BRCA patients with high-risk scores have a worse prognosis. To observe the expression of hub genes at mRNA level in BRCA patients, we analyzed the mRNA levels of TCGA paired samples, and the results suggested that AZU1 and ENO1 were not significantly different between tumor tissues and normal tissues (Figure 7C, D), while LTF was significantly downregulated and LCP1 was upregulated (Figure 7A, B). Similarly, similar results were obtained in the unpaired samples (Figure 7E–H). In addition, we used the in breast tumor and normal tissue derived from LTF, LCP1, AZU1, and ENO1 differences in total protein expression data analysis. The total protein levels of LTF, LCP1, AZU1, and ENO1 were all shown to be downregulated in tumor tissues. To further understand the expression of key genes in different tumor grades, we obtained total protein levels from the Clinical Proteomic Tumor Analysis Consortium (CPTAC) for BRCA patients at various stages. The findings imply that LTF, AZU1, and ENO1 are related to tumor grade (Figure 8E–H), while LCP1 is not substantially different among tumor stages (Figure 8F). To further validate the difference in hub genes’ expression between BRCA patients and normal controls, we downloaded the immunohistochemical results of hub genes from normal and tumor tissues using the HPA Database. The results suggested that LTF, LCP1 and AZU1 were undetectable (Figures 9A,C,D) and ENO1 was significantly reduced in BRCA patients (Figure 9B). Moreover, GeneMANIA database was powered to construct protein-protein interaction networks of LTF, LCP1, AZU1 and ENO1 (Figure 10). The results suggested that the four hub genes may be involved in promoting protein synthesis, myeloid leukocyte differentiation, defense response to bacterium, human immune response, *etc.*

## 4 Discussion

Neutrophil extracellular traps are produced by neutrophils by expelling citrullinated histone H3, myeloperoxidase and other intracellular molecules. Since it was discovered in 2004, a large number of literatures about the formation mechanism of NETs and its relationship with innate immunity and inflammation have emerged (Tan et al., 2021). NETs not only has antibacterial effect, but also may damage organs or tissues due to overexpression. NETs is not only a host defense response, but also plays an important role in infectious and non-infectious diseases (Leliefeld, et al., 2015; Pruchniak, et al., 2019).

In addition, NETs can be found in human and various animal malignancies, such as breast cancer, gastric cancer, colorectal cancer and liver cancer. With the research and deepening of the relationship between NETs and tumor, NETs has been confirmed to be related to tumor invasion, metastasis and immune escape of tumor cell. Among many tumor cells, neutrophils play a role in promoting tumor progression in animal models and cancer patients (Kaltenmeier, et al., 2021). Moreover, and NETs also directly affect tumor related immune microenvironment (Teijeira, et al., 2021). NETs in serum can indicate the occurrence of liver metastasis of early breast cancer (Yang et al., 2020). It is a vicious circle model that metastatic breast cancer cells induce neutrophils to form NETs, which further promotes the growth of tumor cells in target organs (Liang, et al., 2021).

In our study, we found for the first time a BRCA clustering classification method for NETs-related genes. The risk score calculated based on the expression level of related genes can predict whether they will respond to immunotherapy, which has important implications for the selection of treatment modalities for breast cancer patients. Although many studies have confirmed that high NETs expression indicates poor tumor prognosis (Snoderly, et al., 2019; Martins-Cardoso, et al., 2020), the high expression of 22 NETs-related genes in this study suggests that BRCA patients respond better to immunotherapy, which is not contrary to the evidence of previous studies. The reason is not all of 22 NETs-related genes in this study promote the release of NETs, which implies that the NETs high subgroup do not release more NETs than the NETs low subgroup. Specifically, TCGA cohorts were divided into two subgroups (C1 and C2) based on PAM clustering of Net-related genes. Subsequently, heat maps of the two subpopulations in C1 showed higher expression of Nets-related genes in C2. Therefore, we define the C1 cluster as the NETs low group and the C2 cluster as the NETs high group. It is worth mentioning that NET high here is intended to replace cluster 2 and does not mean that NET high promotes NETs formation or related NETs protein secretion. The same goes for NET low. From this perspective, we do not think the results of this study contradict previous studies. Of note, the risk signature model constructed by different NETs-related gene sets may show different results.

22 NETs-related genes in our research were summarized from previous literature. Firstly, we analyzed the expression profiles of 22 NETs related genes in BRCA and normal tissues and found that most of the genes were downregulated in BRCA while there were also a small proportion of genes that were highly expressed in BRCA. Secondly, consensus clustering analysis was utilized to divide BRCA patients into two clusters (C1 and C2), which respectively represents the NETs low group and NETs high group. Moreover, KEGG and GO were applied to analyze the functional enrichment of DEGs between the two subgroups, and GSEA was used to analyze the enriched related signaling pathways. Reports have suggested that NETs formation by neutrophils in the tumor microenvironment plays an active tumor-promoting role during disease progression (Tohme, et al., 2016; van der Windt, et al., 2018; Wu, et al., 2019).

NETs can also activate the NF-κB signaling pathway to directly stimulate the proliferation of tumor cells (Sangaletti, et al., 2014). To observe the differences of tumor immune cell infiltration in different NETs subgroups, we search for the tumor microenvironment landscape of BRCA patients, utilizing ESTIMATE and CIBERSORT to assess the level of BRCA tumor associated immune infiltration and immune scores and also further analyzed immune checkpoint and HLA related genes. Waterfall plot was generated for visualization by analyzing the Somatic mutation data. Additionally, we screened out 4 genes related to prognosis by LASSO Cox regression analysis. And then a risk signature model based on 22 NETs-related genes was established and validated. Besides, a riskscore *versus* immune cell correlation scatter plot was generated to visualization. Notably, we also utilized the TIDE database to predict BRCA response to immunotherapy. As a result, we found that a lower risk score predicted a better prognosis and a more beneficial response to immunotherapy, which suggests that risk scores can be used to cluster BRCA patients and guide clinical treatment strategies.

In conclusion, this study establishes and validates a NETs-related stratification system that is beneficial for predicting clinical outcomes and guiding immunotherapy for BRCA patients, which is particularly necessary for individualized treatment of BRCA patients.

Nonetheless, there are some limitations to the research. Firstly, the BRCA samples included in this study were all types of breast cancer. As different types of breast cancer need different clinical treatments, there will be heterogeneity of BRCA samples in the research, which will affect the accuracy of the results. Secondly, there was no obvious difference in the prognosis between two NETs subgroups because the sample of GSE21653 is not enough. Thus, larger cohort study is needed to validate the accuracy of this stratification system and risk model for NETs in the future. Last but not least, more experiments *in vivo* and *in vitro* should be performed to confirm the feasibility of the research.

## 5 Conclusion

In this study, we found that two distinct breast cancer subtypes (NETs high group and NETs low group) could be obtained by PAM clustering method. We discovered significant prognostic differences between the two subgroups (NETs low indicated poor prognosis) and performed KEGG and GO analyses. In addition, a risk score model based on NETs gene set was established by LASSO regression analysis. Notably, we also found that the riskscore was associated with the response to immunotherapy, suggesting that the riskscore can be used to predict whether a patient will respond to immunotherapy. Therefore, our study provides some useful perspectives for future breast cancer research and clinical treatment.

## Data Availability

The original contributions presented in the study are included in the article/Supplementary Material, further inquiries can be directed to the corresponding author.
