# Supplementary material for: Prediction of prognosis and immunotherapy response in breast cancer based on neutrophil extracellular traps-related classification
Source: Front Mol Biosci. 2023 May 26;10:1165776. doi: 10.3389/fmolb.2023.1165776 (PMC10250592; doi:10.3389/fmolb.2023.1165776)
Supplement: Supplementary file 1 [file Table2.DOCX]

Table S1: Clinical baseline information of TCGA-BRCA

| Characteristic | levels | Overall |
| --- | --- | --- |
| n |  | 986 |
| Sex, n (%) | FEMALE | 977 (99.1%) |
|  | MALE | 9 (0.9%) |
| Event, n (%) | Alive | 852 (86.5%) |
|  | Dead | 133 (13.5%) |
| T, n (%) | T1 | 259 (26.3%) |
|  | T2 | 567 (57.5%) |
|  | T3 | 125 (12.7%) |
|  | T4 | 32 (3.2%) |
|  | TX | 3 (0.3%) |
| N, n (%) | N0 | 461 (46.8%) |
|  | N1 | 329 (33.4%) |
|  | N2 | 111 (11.3%) |
|  | N3 | 68 (6.9%) |
|  | NX | 17 (1.7%) |
| M, n (%) | M0 | 818 (83.5%) |
|  | M1 | 19 (1.9%) |
|  | MX | 143 (14.6%) |
| Stage, n (%) | Stage I | 166 (17%) |
|  | Stage II | 560 (57.4%) |
|  | Stage III | 223 (22.8%) |
|  | Stage IV | 17 (1.7%) |
|  | Stage X | 10 (1%) |
| Age, median (IQR) |  | 58 (48, 67) |
| OS, median (IQR) |  | 848 (442.25, 1679.75) |
